# Supplementary material for: Overexpression generates aberrant distribution of endocytic regulators - the case of the Rab11/LAMP1 compartment
Source: PLoS One. 2026 Apr 22;21(4):e0346157. doi: 10.1371/journal.pone.0346157 (PMC13102219; doi:10.1371/journal.pone.0346157)
Supplement: S1 Fig — (PDF) [file pone.0346157.s001.pdf]

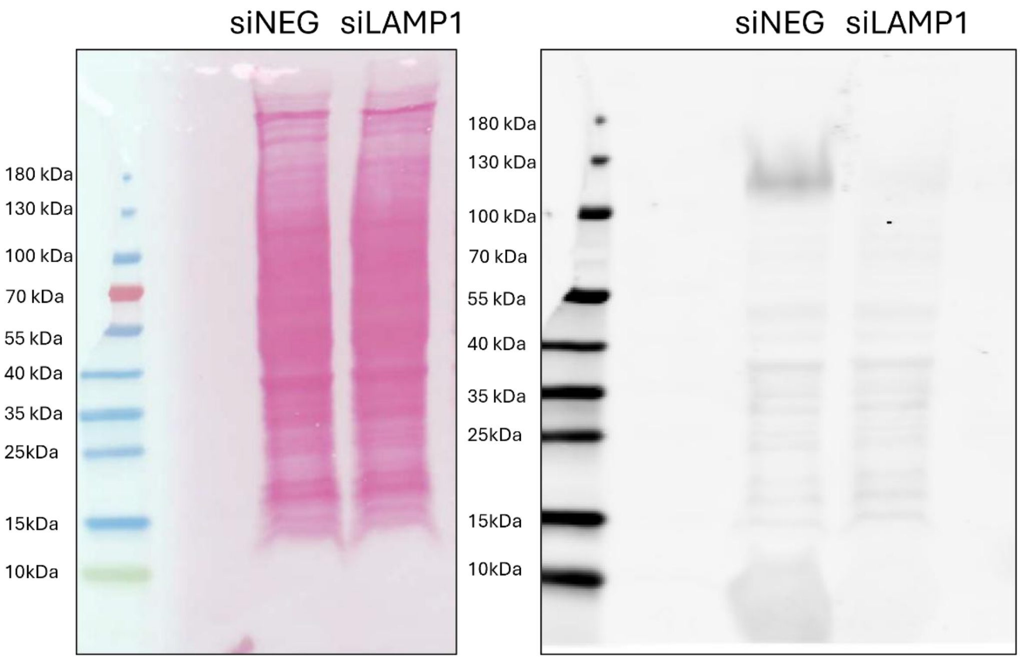


Raw images for Fig 2 A.
Left blot, Ponceau staining. Right blot, LAMP1 staining, 1:1000 dilution of commercial antibody.

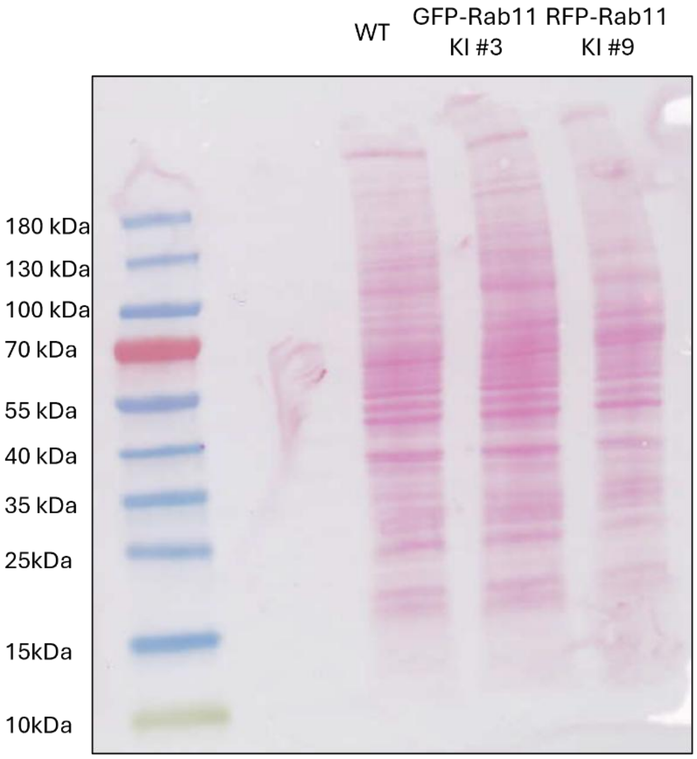


Ponceau S Staining for Fig 2 C.

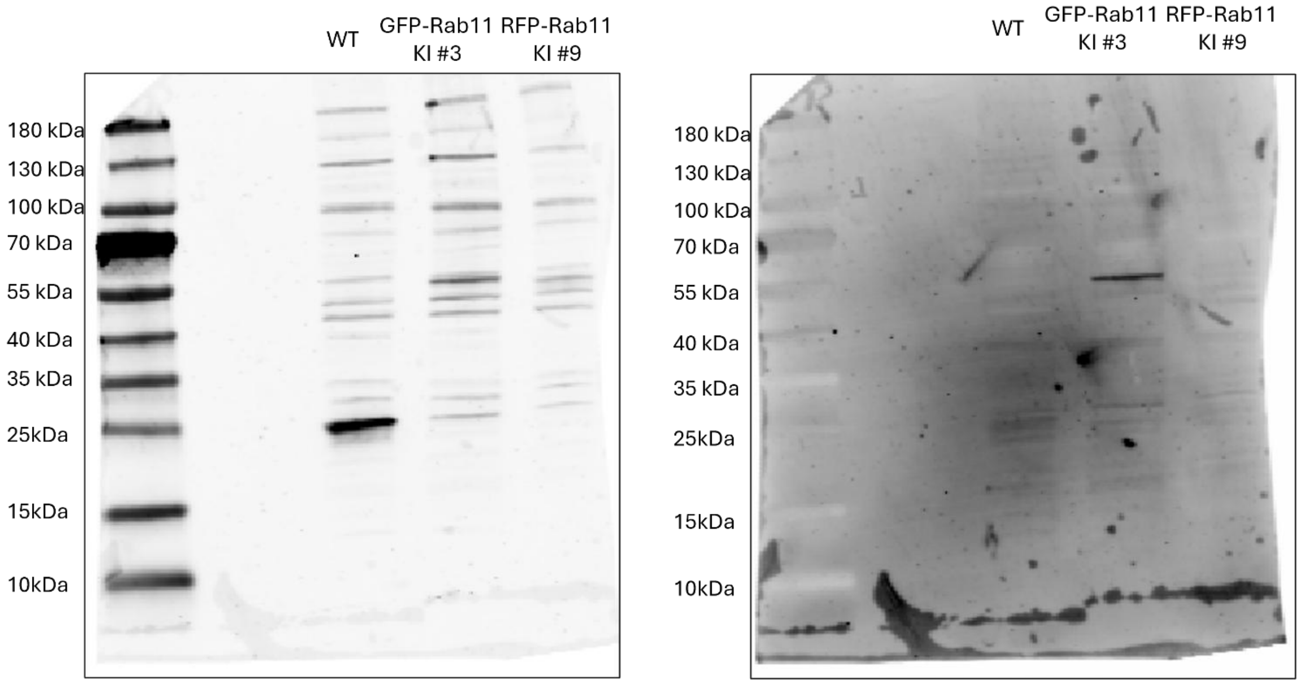


Raw images for Fig 2 C.
Left blot, Rab11 staining, 1:1000 dilution of commercial antibody. Right blot, GFP staining, 1:1000 dilution of commercial antibody.

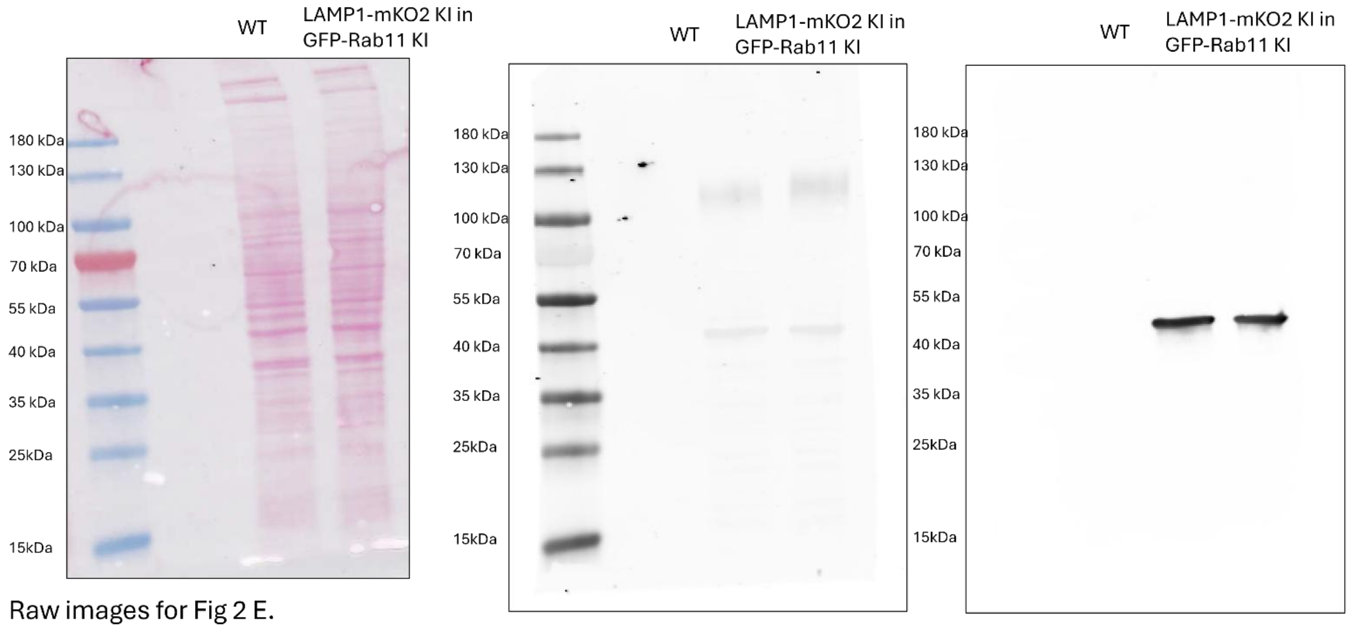


Raw images for Fig 2 E.
Left blot, Ponceau S staining. Middel blot, LAMP1 staining, 1:1000 dilution of commercial antibody visualized with AlexaFluor680. Right blot, β-actin staining on the same membrane, 1:1000 dilution of commercial antibody visualized with AlexaFluor800.
