# Supplementary material for: Overexpression generates aberrant distribution of endocytic regulators - the case of the Rab11/LAMP1 compartment
Source: PLoS One. 2026 Apr 22;21(4):e0346157. doi: 10.1371/journal.pone.0346157 (PMC13102219; doi:10.1371/journal.pone.0346157)
Supplement: S2 Fig — (PDF) [file pone.0346157.s002.pdf]

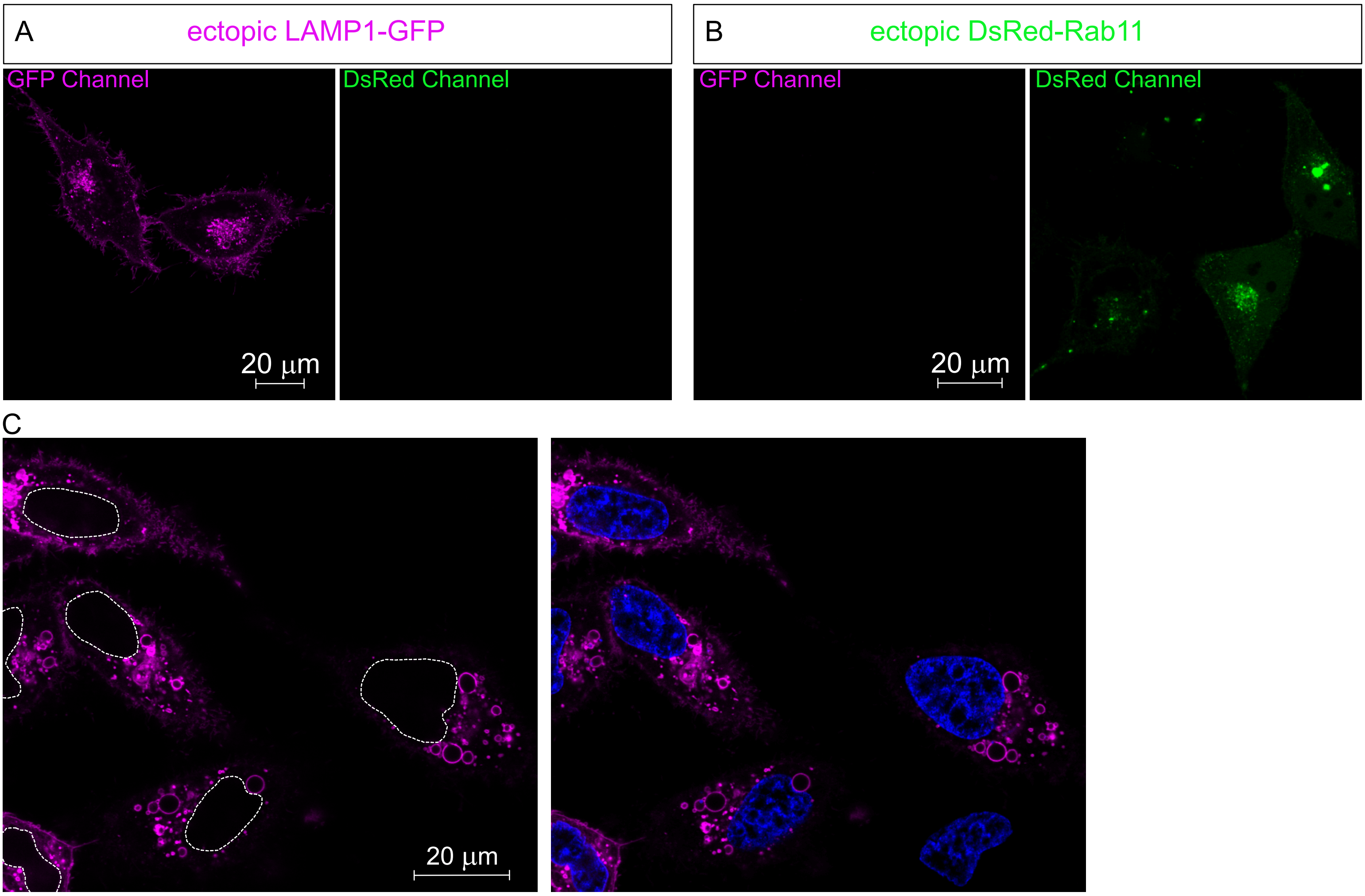

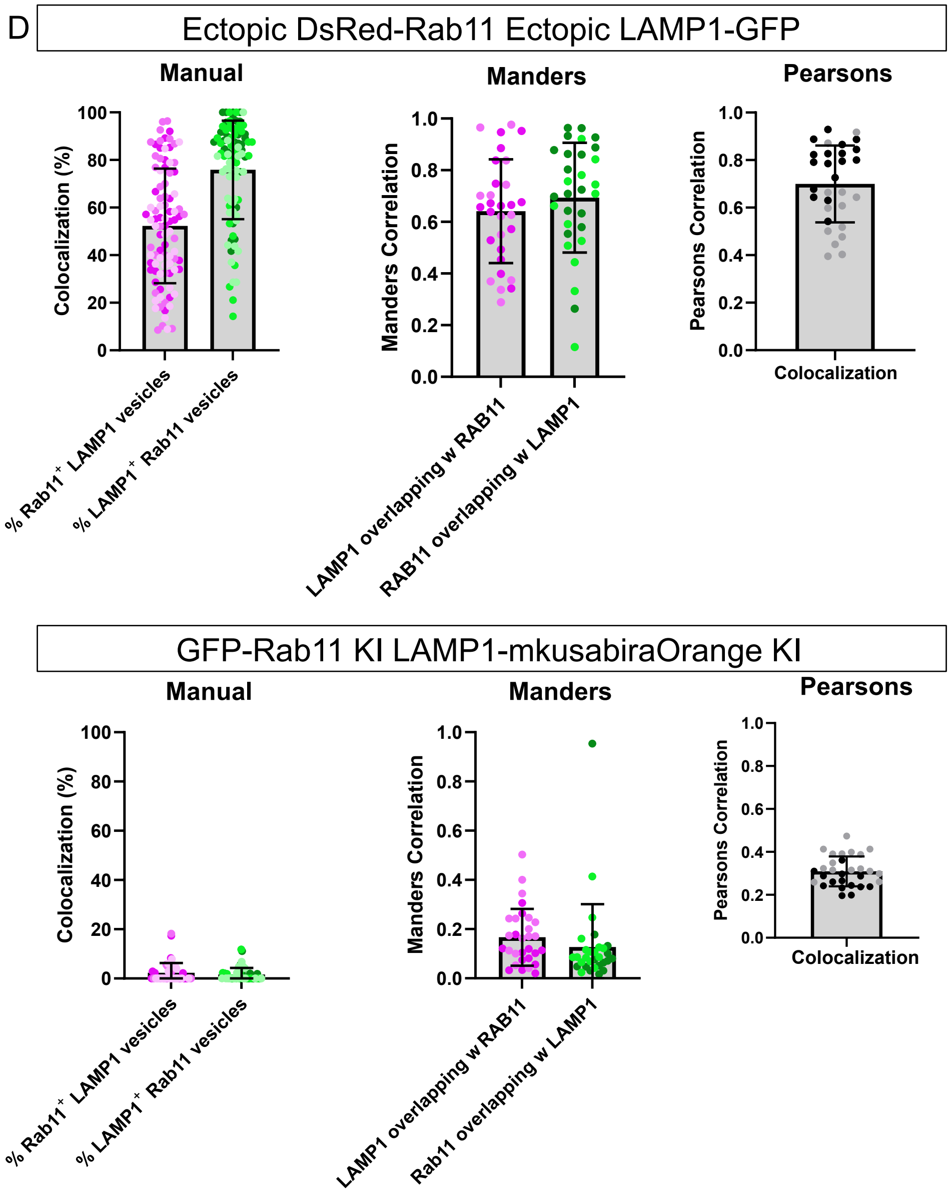

S2 Fig: Additional controls.
A-B No bleed through between imaging acquisition channels Cells expressing LAMP1-GFP (panel A) or DsRed-Rab11 (panel B) were imaged using both the GFP- and the DsRed channels. Scalebar: 20 µm. **C Nuclei can be accurately located in the absence of Hoechst staining** In the left panel, the position of the nuclei was manually drawn on images where the Hoechst staining was disabled and then compared (right panel) with the images where the Hoechst signal was turned on. The experimenters were not allowed to see the Hoechst staining when they drew the outlines of the nuclei. Scalebar: 20 µm.
D Comparison of colocalization methods The manual quantification data are those shown in the main figures. For Manders and Pearson analyses, 30 cells were scored from two independent experiments. The bars in the graph represent the mean and the error bars correspond to the standard deviation.
