# Supplementary material for: Overexpression generates aberrant distribution of endocytic regulators - the case of the Rab11/LAMP1 compartment
Source: PLoS One. 2026 Apr 22;21(4):e0346157. doi: 10.1371/journal.pone.0346157 (PMC13102219; doi:10.1371/journal.pone.0346157)
Supplement: S3 Fig — (PDF) [file pone.0346157.s003.pdf]

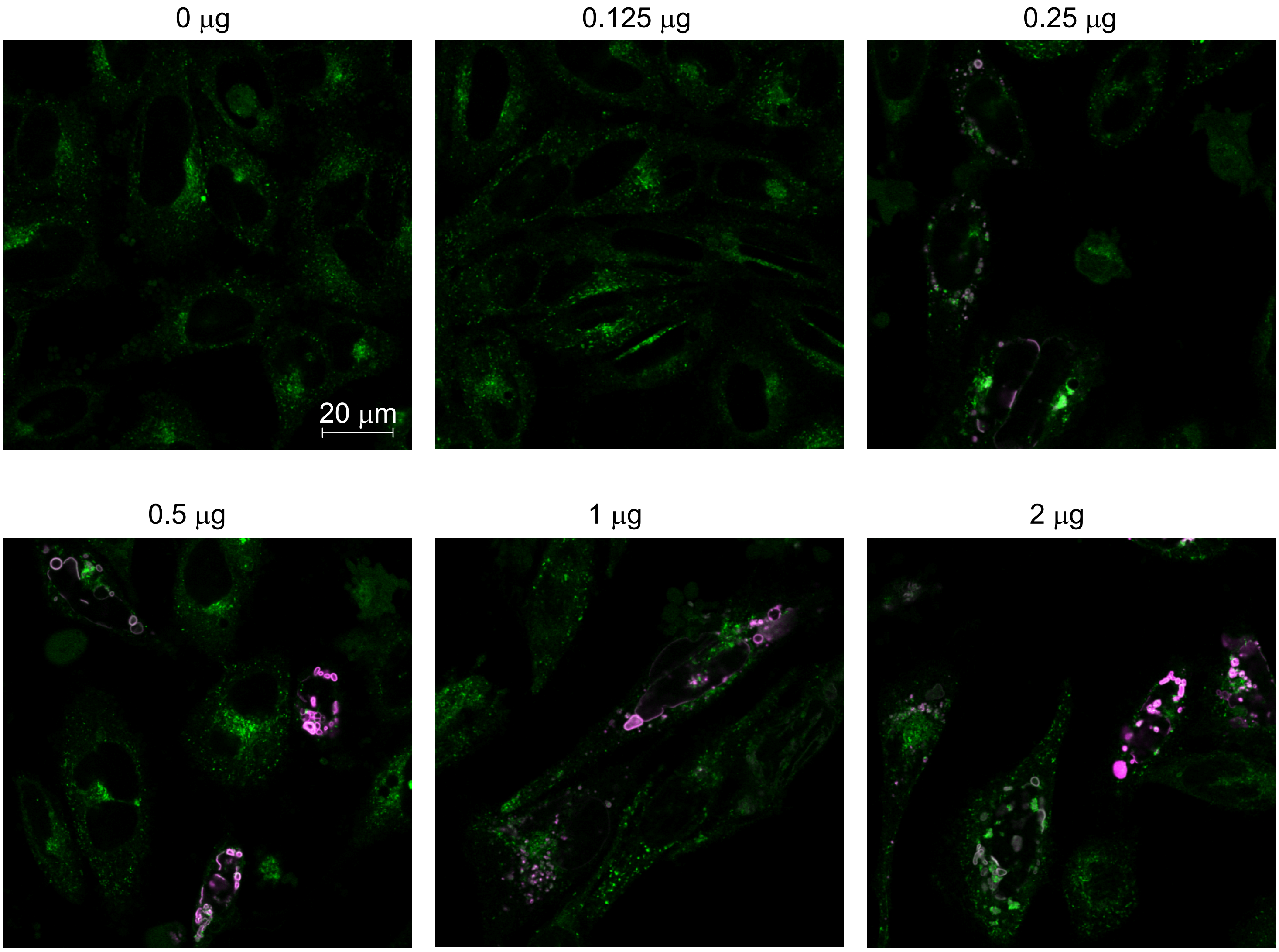

S3 Fig: Titration of DNA for ectopic expression.
Ectopic LAMP1-DsRed was expressed in GFP-Rab11 KI cells using the indicated DNA amounts ranging from 0 μg to 2 μg. LAMP1 is shown in magenta, Rab11 is shown in green; scalebar: 20 μm. Imaging settings were kept the same for all conditions.
