# Supplementary material for: Overexpression generates aberrant distribution of endocytic regulators - the case of the Rab11/LAMP1 compartment
Source: PLoS One. 2026 Apr 22;21(4):e0346157. doi: 10.1371/journal.pone.0346157 (PMC13102219; doi:10.1371/journal.pone.0346157)
Supplement: S4 Fig — (PDF) [file pone.0346157.s004.pdf]

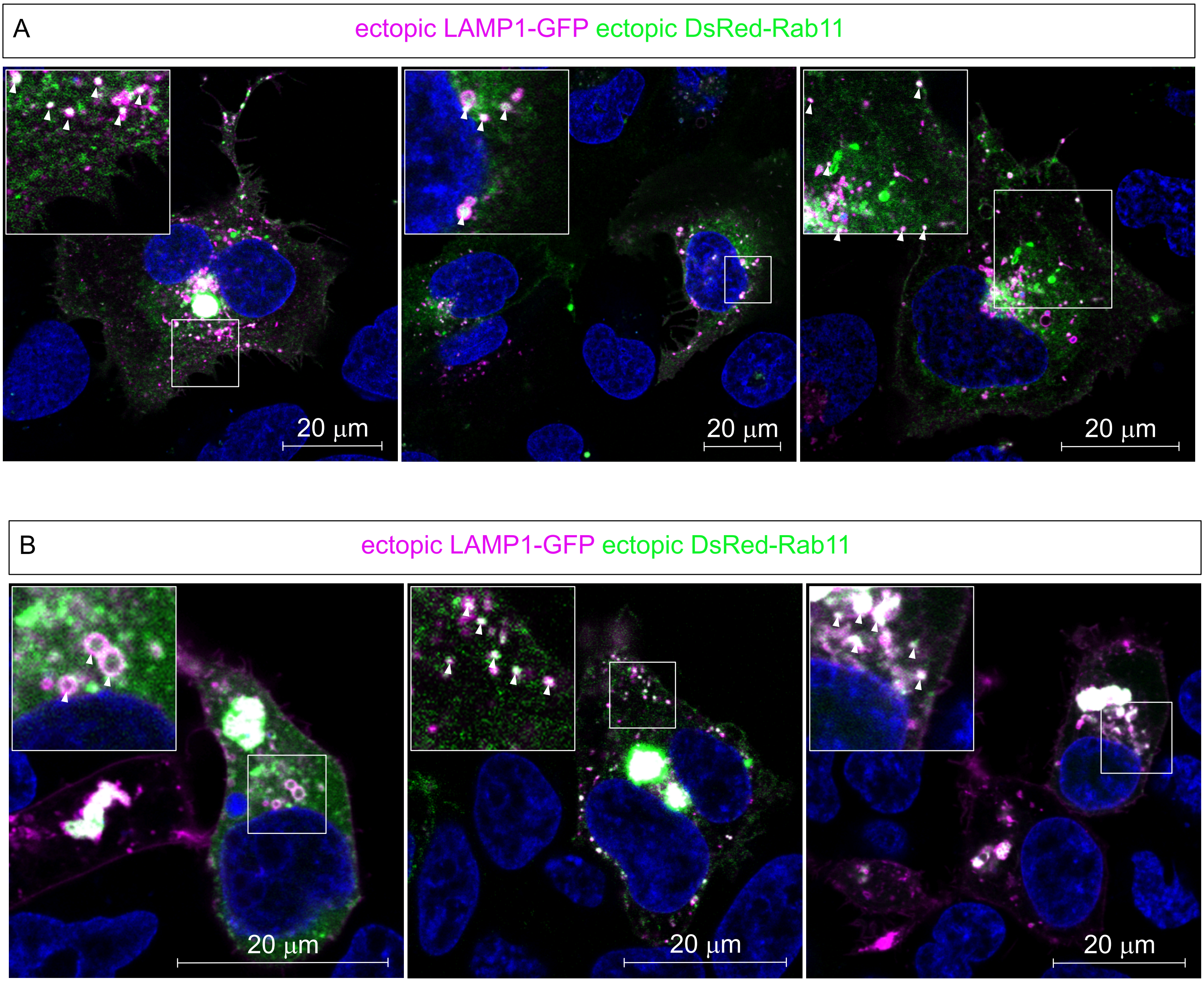

S4 Fig: Colocalization of ectopic Rab11 and LAMP1 in additional cell lines.
A-B Representative pictures of U2OS cells (panel A) or HEK293T (panel B) transfected with DsRed-Rab11 and LAMP1-GFP. The magenta pseudocolour shows LAMP1, the green pseudocolour, Rab11, and the white pseudocolour, colocalization. Scalebars: 20 μm. The zoomed areas are indicated by squares. Arrowheads point to structures where colocalization occurs.
