# Supplementary material for: Overexpression generates aberrant distribution of endocytic regulators - the case of the Rab11/LAMP1 compartment
Source: PLoS One. 2026 Apr 22;21(4):e0346157. doi: 10.1371/journal.pone.0346157 (PMC13102219; doi:10.1371/journal.pone.0346157)
Supplement: Attachment — Submitted filename: Response to Reviewers.docx [file pone.0346157.s007.docx]

PONE-D-25-49367

Overexpression generates aberrant distribution of endocytic regulators - the case of the Rab11/LAMP1 compartment

PLOS One

Dear Dr. Widmann,

Thank you for submitting your manuscript to PLOS ONE. After careful consideration, we feel that it has merit but does not fully meet PLOS ONE’s publication criteria as it currently stands. Therefore, we invite you to submit a revised version of the manuscript that addresses the points raised during the review process.

Please submit your revised manuscript by Jan 23 2026 11:59PM. If you will need more time than this to complete your revisions, please reply to this message or contact the journal office at plosone@plos.org. When you're ready to submit your revision, log on to https://www.editoriatmanager.com/pone/ and select the 'Submissions Needing Revision' folder to locate your manuscript file.

Please include the following items when submitting your revised manuscript:

- A rebuttal letter that responds to each point raised by the academic editor and reviewer(s). You should upload this letter as a separate file labeled 'Response to Reviewers'.
- A marked-up copy of your manuscript that highlights changes made to the original version. You should upload this as a separate file labeled 'Revised Manuscript with Track Changes'.
- An unmarked version of your revised paper without tracked changes. You should upload this as a separate file labeled 'Manuscript'.

If you would like to make changes to your financial disclosure, please include your updated statement in your cover letter. Guidelines for resubmitting your figure files are available below the reviewer comments at the end of this letter.

If applicable, we recommend that you deposit your laboratory protocols in protocols.io to enhance the reproducibility of your results. Protocols.io assigns your protocol its own identifier (DOI) so that it can be cited independently in the future. For instructions see https://journals.plos.org/plosone/s/submisslon-guidetines#loc-laboratory-protocols. Additionally, PLOS ONE offers an option for publishing peer-reviewed Lab Protocol articles, which describe protocols hosted on protocols.io. Read more information on sharing protocols at https://plos.org/protolocs?utm_medium=editorial-email&utm_source=authorletters&utm_campaign=protocols.

We look forward to receiving your revised manuscript.

Kind regards,

Prasanth Puthanveetil
Academic Editor
PLOS One

Journal Requirements:

When submitting your revision, we need you to address these additional requirements.

1. Please ensure that your manuscript meets PLOS ONE's style requirements, including those for file naming. The PLOS ONE style templates can be found at

https://journals.plos.org/plosone/s/file?id=wjVg/PLOSOne_formatting_sample_main_body.pdf and https://journals.plos.org/plosone/s/file?id=ba62/PLOSOne_formatting_sample_title_authors_affiliations.pdf

1. We adapted the style in order to match the template.

2. Thank you for stating in your Funding Statement:

[This work is supported by the Swiss National Science Foundation (grant n° 310030_207464).].

Please provide an amended statement that declares *all* the funding or sources of support (whether external or internal to your organization) received during this study, as detailed online in our guide for authors at https://journals.plos.org/plosone/s/submit-now. Please also include the statement “There was no additional external funding received for this study.” in your updated Funding Statement.

Please include your amended Funding Statement within your cover letter. We will change the online submission form on your behalf.

2. We placed our Funding Statement in the cover letter.

3. Thank you for stating the following in the Acknowledgments Section of your manuscript:

[The laboratory of CW is supported by the Swiss National Science Foundation (grant n° 310030_207464). We are thankful to the Cellular Imaging Facility at the University of Lausanne for the resources provided and their technical help.]

We note that you have provided funding information that is currently declared in your Funding Statement. However, funding information should not appear in the Acknowledgments section or other areas of your manuscript. We will only publish funding information present in the Funding Statement section of the online submission form.

Please remove any funding-related text from the manuscript and let us know how you would like to update your Funding Statement. Currently, your Funding Statement reads as follows:

[This work is supported by the Swiss National Science Foundation (grant n° 310030_207464).]

Please include your amended statements within your cover letter; we will change the online submission form on your behalf.

3. We deleted our Funding Statement from the Acknowledgment section in the main manuscript and added the updated Funding Statement in our cover letter.

4. We note that your Data Availability Statement is currently as follows: [All relevant data are within the manuscript and its Supporting Information files.]

Please confirm at this time whether or not your submission contains all raw data required to replicate the results of your study. Authors must share the “minimal data set” for their submission. PLOS defines the minimal data set to consist of the data required to replicate all study findings reported in the article, as well as related metadata and methods (https://journals.plos.org/plosone/s/data-availability#loc-minimal-data-set-definition).

For example, authors should submit the following data:

- The values behind the means, standard deviations and other measures reported;
- The values used to build graphs;
- The points extracted from images for analysis.

Authors do not need to submit their entire data set if only a portion of the data was used in the reported study.

If your submission does not contain these data, please either upload them as Supporting Information files or deposit them to a stable, public repository and provide us with the relevant URLs, DOIs, or accession numbers. For a list of recommended repositories, please see https://journals.plos.org/plosone/s/recommended-repositories.

If there are ethical or legal restrictions on sharing a de-identified data set, please explain them in detail (e.g., data contain potentially sensitive information, data are owned by a third-party organization, etc.) and who has imposed them (e.g., an ethics committee). Please also provide contact information for a data access committee, ethics committee, or other institutional body to which data requests may be sent. If data are owned by a third party, please indicate how others may request data access.

4. All the raw data used for the generation of our figures are now placed in S5 Table.

5. PLOS ONE now requires that authors provide the original uncropped and unadjusted images underlying all blot or gel results reported in a submission’s figures or Supporting Information files. This policy and the journal’s other requirements for blot/gel reporting and figure preparation are described in detail at https://journals.plos.org/plosone/s/figures#loc-blot-and-gel-reporting-requirements and https://journals.plos.org/plosone/s/figures#loc-preparing-figures-from-image-files. When you submit your revised manuscript, please ensure that your figures adhere fully to these guidelines and provide the original underlying images for all blot or gel data reported in your submission. See the following link for instructions on providing the original image data: https://journals.plos.org/plosone/s/figures#loc-original-images-for-blots-and-gels.

In your cover letter, please note whether your blot/gel image data are in Supporting Information or posted at a public data repository, provide the repository URL if relevant, and provide specific details as to which raw blot/gel images, if any, are not available. Email us at plosone@plos.org if you have any questions.

If the reviewer comments include a recommendation to cite specific previously published works, please review and evaluate these publications to determine whether they are relevant and should be cited. There is no requirement to cite these works unless the editor has indicated otherwise.

5. We generated a single PDF file with all raw images (S1_raw_images.pdf) and stated this in the cover letter.

Additional Editor Comments:

Dear Authors,

Please address the concerns raised by our reviewers and submit your revised version within the allocated time.

Looking forward to your submission.

Thanks,
Handling Editor.

[Note: HTML markup is below. Please do not edit.]

Reviewers' comments:

Reviewer's Responses to Questions

**Comments to the Author**

1. Is the manuscript technically sound, and do the data support the conclusions?

The manuscript must describe a technically sound piece of scientific research with data that supports the conclusions. Experiments must have been conducted rigorously, with appropriate controls, replication, and sample sizes. The conclusions must be drawn appropriately based on the data presented.

Reviewer #1: Yes

Reviewer #2: Yes

Reviewer #3: Yes

Reviewer #4: Yes

Reviewer #5: Partly

2. Has the statistical analysis been performed appropriately and rigorously?

Reviewer #1: Yes

Reviewer #2: N/A

Reviewer #3: Yes

Reviewer #4: N/A

Reviewer #5: No

3. Have the authors made all data underlying the findings in their manuscript fully available?

The PLOS Data policy requires authors to make all data underlying the findings described in their manuscript fully available without restriction, with rare exception (please refer to the Data Availability Statement in the manuscript PDF file). The data should be provided as part of the manuscript or its supporting information, or deposited to a public repository. For example, in addition to summary statistics, the data points behind means, medians and variance measures should be available. If there are restrictions on publicly sharing data—e.g. participant privacy or use of data from a third party—those must be specified.

Reviewer #1: Yes

Reviewer #2: Yes

Reviewer #3: Yes

Reviewer #4: Yes

Reviewer #5: Yes

4. Is the manuscript presented in an intelligible fashion and written in standard English?

PLOS ONE does not copyedit accepted manuscripts, so the language in submitted articles must be clear, correct, and unambiguous. Any typographical or grammatical errors should be corrected at revision, so please note any specific errors here.

Reviewer #1: Yes

Reviewer #2: Yes

Reviewer #3: Yes

Reviewer #4: Yes

Reviewer #5: Yes

5. Review Comments to the Author

Please use the space provided to explain your answers to the questions above. You may also include additional comments for the author, including concerns about dual publication, research ethics, or publication ethics. (Please upload your review as an attachment if it exceeds 20,000 characters)

Reviewer #1: In the current report, Hornfeck et al. studied the subcellular distribution of Rab11 and Lamp1, known to reside in the recycling endosome and late endosome/lysosome, respectively. Not quite surprisingly, the authors found that ectopic overexpression of these proteins, tagged with fluorescent markers, affects their distribution, leading to wrong conclusions about their subcellular colocalization. When both proteins were knocked in to allow endogenous expression, no colocalization was observed.
While the data provided seems rather convincing, the overall information is too limited to justify publication as a Research Article or even Qualitative Research. The authors are encouraged to utilize the double knock-in cells to determine questions regarding these interesting organelles.

We thank Reviewer 1 for having found our manuscript technically sound and supported by the presented data (see boxes ticked in sections 1-4).

6. The main purpose of our study is to highlight artefactual generation of endosomal compartments upon ectopic expression using the Rab11/LAMP1 compartment as a poster child. Hence, we did not aim at describing this artificial compartment functionally but rather at raising awareness for potential pitfalls when characterizing vesicular structures in cells. We believe it would be beyond the scope of the present work to start characterizing the LAMP1/Rab11 artefactual compartment. And again, we question somehow the relevance to doing so.

Reviewer #2: A clear, concise and thorough article that used logical scientific approaches to highlight an important methodological concern in trafficking research. Although it is widely accepted that ectopic protein expression leads to abnormally high protein expression levels and likely alters functional pathways, evidence for this is very rarely published likely due to negative results bias. This article provides novel citable evidence in a commonly used cell model in the field. Publication of this data can benefit relevant groups when designing experiments and considering results as well as prevent other research groups from falsely identifying this now discredited intermediate vesicular compartment.

7. We thank Reviewer 2 for this positive feedback.

An appropriate number of experimental combinations for ectopic and endogenous expression levels was performed to support their conclusion. Strong evidence with robust correlation between images and quantification. Very clear explanation of results and methods which could be easily reproduced. No statistical analysis, but can't identify any need within this study.

Although the evidence here shows ectopic protein expression can have negative consequences, it is common practice to titrate the amount of DNA in ectopic expression experiments to try and achieve less abnormal expression patterns. Optimisation of the DNA concentrations to reduce aberrant colocalization would increase the impact of this research, 2ug is high. Minimally, address this in the article text as a limitation.

8. This remark is well taken and we now include a figure (S3 Fig) showing how DNA amounts used during cell transfection affected colocalization between LAMP1 and Rab11. This figure shows that, starting from 0.25 μg DNA, the first concentration where fluorescent signal above the background can be observed, a similar colocalization between LAMP1 and Rab11 is seen as when higher DNA quantities were used (see lines 100-102 in the track change version).
   We also now mention in the method section, for each plasmid, the amount of DNA used for transfection.

Suggested improvements:
- Fig 1E and 3C are not referred to in text about colocalisation quantification, adding this would be beneficial.

9. This now has been done.

- Line 95: 'with when' remove with

10. We deleted “with” from this sentence.

- Changing the figure order to have 2 C/D above 2A/B would be beneficial to the reader in order of how it is read.

11. We changed the order of Figure 2 and changed the references in the text as well.

Recommended for acceptance after these minor revisions.

Reviewer #3: The authors address the relevance of studying vesicular trafficking at endogenous level and how the overexpression of Rab11 and LAMP1 generates a colocalization that was not present in an endogenous context. Besides, they showed how the approach of an overexpression by using tagged proteins could produce artefactual compartments. Although is a very interesting work, there are some controls and questions that should be addressed.

12. We thank the reviewer for this positive feedback. Below are our answers to the raised comments and questions.

1.- Lane 52, include more recent works in Rab11 trafficking and recycling.

13. We included more recent references for Rab11 and the trafficking and recycling of various cargos (references 5-7).

2.- Lane 53, include recent works for LAMP1 context.

14. We included more recent references for LAMP1 (references 8-10).

3.- Lane 84, include the name of the two fluorescent proteins in this part of the text.

15. Their full names were added.

4.- Lane 86, all Figure 1, include the nuclear staining, either DAPI or Hoechst.

16. As we now show (new Supplemental Figure 2 C), the outline where the nucleus is can be readily identified without Hoechst by recording the position within the cells lacking fluorescence. We did not need therefore to stain the cells with Hoechst to determine the position of the nuclei.

5.- Lane 91, Figure 2C, obtain a housekeeping signal for the Western Blot results, and a contour for each wb result will be fine.

17. We included the Ponceau S staining for the whole protein loading and framed the Western blots.

6.- Lane 96, Figure 2A, include a housekeeping signal for the Western Blot result, I suggest repeating the whole Western Blot by running the protein extracts in the same gel, thus, getting compared signal results.

18. We repeated the whole Western blot, ran the protein extracts in the same gel and in addition added and anti-GFP antibody for the GFP-Rab11 KI cell line.

7.- Lane 332, Figure 3C, look for a better representation of the points in the graphic, because is barely appreciate, most of them are enclose in the 20% range, so, a better distribution of them should be addressed for a better analysis.

19. We added blots with the axis set to 20% to better visualized the distribution of the points in these figures. This is now mentioned in the Figure legend.

8.- Lane 331, figure 2A, get the signal by using a GFP antibody in the Western Blot for the fusion proteins.

20. As we mention for comment #18, we now include an additional staining of the blot using an anti-GFP antibody. Please note that the GFP antibody does not recognize RFP.

9.- Do the authors think that by using an inducible system under the control of Doxocycline for example, it will be possible to reach good results by using tagged proteins? For vesicular traffic studies?

21. Indeed, alternative approaches to reduce the amount of overexpressed protein might ameliorate some of the adverse effects due to overexpression. For example, the use of inducible systems or weak promoters might allow low ectopic expression to be achieved. This would have to tested experimentally though. On the other hand, diminishing the amount of expression plasmids with strong promoters during the transfection procedures may not prevent the formation of aberrant vesicular compartments as shown in supplementary Figure 3).

Reviewer #4: S. Hornfeck et al. present an interesting and important manuscript, as it highlights the often overlooked risk of creating artifacts through the overexpression of proteins and/or through tagging them with fluorophores like GFP.

They set out to investigate the role of Rab11 in the internalization, recycling or degradation processes. They convincingly demonstrate that, when investigating this question using the common approach of overexpressing labelled fusion proteins, artifacts can arise that do not reflect the situation of these proteins under physiological expression levels. Upon overexpression they observed colocalization of Rab11 and LAMP1 in many vesicles, suggesting the existence of an intermediate compartment of recycling endosomes and lysosomes. However, they did not observe this at physiological expression levels.

22. We thank the reviewer for the positive feedback and for the comments raised.

In general, the experiments were well conducted and included appropriate controls. However, a few points should be addressed:
P12, line 110: clarify in which of the images in Fig. 1 LAMP-1 is ectopically expressed, as it is not the case in Fig.1B

23. We added the clarification that LAMP1 is ectopically expressed in Figure 1A, C, and D.

Page 12, line 115: should be GFP-Rab11 KI (makes more sense and is so written in the Fig. 3B)

24. We changed RFP-Rab11 KI to GFP-Rab11 KI.

Page 12, line 117 Transfer “Figure 2E” to line 115 after “Lamp1 locus” as Figure 2E does not show a double expressing cell line.

25. We transferred the reference for Figure 2E to after “LAMP1 locus”

Page 12, line 117. Replace “Figure 2E” by “Figure 3B”. “Figure 3B” can now be removed from line 118.

26. We replaced the Figure references and removed Figure 3B from this line.

Page 16, lines 192 -194: this is not a full sentence, verb is missing (contained?)

27. We replaced “containing” by “contained”.

Page 16, lines 201: word is missing

28. We added the missing word (fluorescent tag) in the manuscript.

Among the antibodies listed in “Materials and Methods”, it is not clear for all of them where they were used:
In Fig. 1B the FITC-anti-mouse obviously was used in combination with ectopic DsRed-Rab11 for labelling of endogenous LAMP1. Where was the listed AlexaFluor647 conjugated anti-mouse antibody used at 1:500 for immunofluorescence (page 16, line 175)?

29. The AlexaFluor647 conjugated anti-mouse antibody was used at 1:500 in Figure 3A. We clarified this in the figure legend (see line 162 in the track change version).

Page 16, line 173: “The Cy5 conjugated anti-Rabbit antibody was used at 1:500 for immunofluorescence“. Where?

30. The Cy5 conjugated anti-Rabbit antibody was indeed not used in this paper. We deleted it from the methods. We are sorry for this mistake.

It would indeed have been even more convincing if both endogenous proteins Rab11 and LAMP-1 - without any fused fluorescent tags - had been detected only by antibodies. That might have been the right antibody to do it.

31. We agree that using two antibodies instead of fused fluorescent tags would have been more ideal. Unfortunately, we could not find an antibody against Rab11 which is working reliably in immunofluorescence (IF). The antibody we tested for Rab11 was still staining structures in Rab11 silenced cells to similar extent as in the control cells. In our hands, it was hard to find reliable antibodies against Rab proteins that could work in IF.

Fig. 1: Could the authors comment on why the vesicles in C and D look so different, even though the main difference is only in the colour of the fluorophores. Off-target effects? Did all clones look like this?

32. The ectopic expression of LAMP1 changes the appearance and size of Rab11-positive vesicles in both KI cell lines. In the GFP-Rab11 KI cell line, the overexpression of LAMP1-DsRed induces a more pronounced increase in the size of vesicles compared to the RFP-Rab11 KI cells overexpressing LAMP1-GFP. The reasons for this are unclear at the present time. However, there were no difference between these two cell lines in terms of the size of the Rab11-positive vesicles (see Figure below). Off-target effects are unlikely in our opinion because no other GFP-positive bands than the one corresponding to GFP-Rab11 were detected in the Western blot shown in Figure 2. This indicates that GFP insertion only occurred at the expected locus in the Rab11 gene.

Page 17, line 201: Could the authors elaborate on how many cells successfully replaced the wild type with the fusion protein and whether the phenotype was affected through off-target effects. Which clones where selected for the experiments?

33. Out of the 9 GFP-Rab11 KI clones isolated after flow cytometry sorting, only one (clone #3) lost all wild-type alleles, i.e. no detection of a WB band corresponding to the wild-type untagged Rab11 protein, but only a band corresponding to GFP-Rab11. Similarly, only 3 clones out of the 15 RFP-Rab11 KI clones had all its Rab11 alleles successfully targeted. The paper describing the methodology we used (Manna et al. 2019) stated that approximately 0.5% of the transfected cells will incorporate the knock-in fluorescent protein. In both the GFP-Rab11 and RFP-Rab11 selected KI cell lines, the Rab11 vesicles appear similar (see Figure above). Their morphological phenotypes were apparently not altered (size, shape, or adhesion properties). The only difference we noted is that they grew slightly less fast that the wild-type HeLa cells.

If western blot was standard procedure, why isn’t there one shown for the LAMP1-mKusabiraOrange2 KI in Fig. 2?

34. We could not specifically detect the presence of tagged construct because of the lack of a commercial antibody that would be specific for mKusabiraOrange2. We do now however provide a Western blot comparing wild-type HeLa cells and the GFP-Rab11 and LAMP1-mKusabiraOrange2 double KI (Figure 2E). This blot shows a upward shift in the molecular weight of LAMP1-positive band in the double KI cell compared to the wild-type cells, consistent with the notion LAMP1 is bearing the fluorescent protein in the double KI cells. Evaluation of LAMP1 in Western blots is complicated though by the fact that LAMP1 is heavily post-translationally modified in cells as can be seen by the size of the detected LAMP1 bands that are in the 110-140 kDa range while the theoretical size of the protein is only 45 kDa (see this [Cell Signaling webpage](https://www.cellsignal.com/products/primary-antibodies/lamp1-c54h11-rabbit-monoclonal-antibody/3243?srsltid=AfmBOoq4Dtm5QCPZFrqYWmClRgFEBuA8_YcBuazFin_qFnS0FBggCH7v)). Note also that we confirmed that the observed LAMP1 signal in confocal microscopy in the double KI cells is specific (Figure 2F).

Reviewer #5: General Comments
This manuscript addresses an important methodological issue in cell biology—the widespread reliance on transient overexpression of fluorescently tagged endosomal markers. By systematically comparing the colocalization patterns of Rab11 and LAMP1 under overexpression versus endogenous expression, the authors demonstrate that high levels of ectopic expression can generate artefactual endosomal compartments.
However, the study relies almost entirely on qualitative imaging and lacks sufficient quantitative and mechanistic depth to fully substantiate its conclusions. Below, I outline major and minor points that should be addressed before publication.

35. As we show in the new Supplementary Figure 2, the automated methods to record colocalization, such as Pearsons and Manders, more or less reflect the manually measured colocalization. They are however less precise in capturing slight colocalization changes (for example see the upper left panel in Supplementary Figure 2D). Moreover, these automated techniques overestimate the background signal (see the lower left panel in Supplementary Figure 2D). We did not investigate the mechanisms leading to the formation of the artefactual LAMP1/Rab11 compartment for reason explained in our response to comment 6 of Reviewer 1.

Major Comments

1. Quantitative Colocalization Analysis
• The quantification of Rab11/LAMP1 overlap is currently based on manual visual scoring. This approach is inherently subjective and insufficient for publication.
• The authors should apply standard quantitative methods such as Pearson’s correlation coefficient, Manders’ coefficients, or object-based colocalization analysis, with proper controls.

36. Pearsons correlation coefficient and the Manders correlation is now shown in the Supplementary Figure 2 for two of the experimental conditions (both LAMP1 and RAB11 ectopic and both LAMP1 and RAB11 as KI). As explained in our response to comment #35, manual quantitation is much more precise in our hands and this is why we favoured this type of quantitation throughout the paper.

• Include GFP/DsRed-only controls to exclude bleed-through and aggregation artefacts.

37. This is now done in new Supplementary Figure 2 (A and B).

• Provide the number of cells and vesicles analyzed, the statistical tests applied, and the variation across replicates (e.g., standard deviation or SEM).

38. The number of cells was already included in the legends of the corresponding Figures. The number of vesicles analysed are now also mentioned. In our opinion, and this is apparently the case for the other reviewers, we do not think that there is a need to provide statistical analyses for the data we provide. We do provide however, all means and standard deviations in S5 Table.

2. Mechanistic Explanation for Artefactual Colocalization
• The manuscript convincingly shows that overexpression causes Rab11 and LAMP1 to colocalize but does not explore the underlying mechanism.
• The authors should at least discuss possible explanations, such as:
o Overexpression saturating Rab effectors or competing for membranes.
o Altered membrane identity due to overloading of Rab GTPases.
o Non-physiological aggregation of tagged proteins.

39. As this is a case report for the problem arising from ectopic expression, the scope of this paper was not to explore the underlying mechanism. See also our response to #36.

• Would using alternative or weaker promoters that reduce expression levels mitigate this artefact?

40. Please see our answer to comment #21 where we discuss alternatives such as the use of weaker promotors in the plasmids used for transfection.

• Does the Rab11/LAMP1-positive compartment have a functional impact on cargo trafficking?

41. This is an interesting question but since we described here that this compartment is an artefact based on overexpression, we assume it has no functional impact on physiological cargo trafficking. Therefore, we decided not to following this line of investigation.

3. Discussion Depth and Context
The discussion reiterates known concerns about overexpression but could go further. The authors should relate their findings to Rab cascade models (Rab5–Rab7–Rab11 transitions) and mechanisms of endosomal identity maintenance. It would also be valuable to highlight how this artefact may have influenced previous studies and to discuss alternative approaches to overcome these limitations.

42. The concerns about overexpression might be known for several fields but to our knowledge this has not been reported in the context of vesicular trafficking. How this artefact may have influenced previous studies is difficult to assess, because when appropriate controls are not made, it is hard to predict whether the observed effects are due to overexpression or not. Therefore, we did not want to speculate on this topic. In our discussion, we mention that key experiments should preferably be validated under endogenous conditions.

4. Cell-Type Specificity
All experiments are performed in HeLa cells. A note on whether this artefact generalizes to other cell types (e.g., epithelial or neuronal cells) would strengthen the study’s impact.

43. We now included representative pictures of U2OS and HEK239T ectopically expressing DsRed-Rab11 and LAMP1-GFP to show that there is also artefactual colocalization between Rab11 and LAMP1 in these cells (Supplementary information S4 Fig and discussion;see lines 179-191 in the track change version).

Minor Comments
1. Replace phrases such as “a novel compartment” with “an artefactual compartment generated by overexpression” throughout the text.

44. We replaced “a novel compartment” with your suggestion.

2. Specify whether Rab11 and LAMP1 constructs were N- or C-terminally tagged.

45. As implied in the notation of the plasmids, DsRed-hRab11 was N-terminally tagged and all plasmids using LAMP1 were C-terminal tagged. We better specified this in the method section.

3. In the Methods section, specify:
a. How vesicles were identified (thresholding criteria, size filters).

46. We included the reference to a paper where this is described in detail in the method section as well as the cross-reference for the validation by Manders and Pearsons (Figure S2).

b. Image acquisition parameters (z-step size, laser power, pixel dwell time, gain).

47. The imaging parameters such as the pixel size and the line averaging was already mentioned in the methods. Since we did not acquire any z-stacks, there is no z-step size here. We added a comment for the laser power and gain in the Methods. We added the information for Pixel dwell time in the Methods.

4. Ensure consistent gene/protein notation (e.g., Rab11A vs. Rab11).

48. We ensured consistent protein notation by removing the only occurrence of Rab11A in the antibody part of the Method section.

6. PLOS authors have the option to publish the peer review history of their article (what does this mean?). If published, this will include your full peer review and any attached files.

If you choose “no”, your identity will remain anonymous but your review may still be made public.

**Do you want your identity to be public for this peer review?** For information about this choice, including consent withdrawal, please see our Privacy Policy.

Reviewer #1: No

Reviewer #2: No

Reviewer #3: No

Reviewer #4: No

Reviewer #5: **Yes:** Bhuvanasundar Ranganathan
